# Analysis of the *MYB* gene family in tartary buckwheat and functional investigation of *FtPinG0005108900.01* in response to drought

**DOI:** 10.1186/s12870-024-06019-y

**Published:** 2025-01-07

**Authors:** Jinbo Li, Xin Yang, Bianling Tian, Tian Tian, Yu Meng, Fei Liu

**Affiliations:** 1 Life Science College, Luoyang Normal University, Luoyang, 471934 China; 2 State Key Laboratory of Crop Stress Adaption and Improvement, School of Life Sciences, Henan University, Kaifeng, Henan 475004 China; 3 College of Landscape and Travel, Hebei Agricultural University, Baoding, 071001 China

**Keywords:** *Fagopyrum tataricum*, Drought, Transcription factor, Lignin, Transcriptome

## Abstract

Tartary buckwheat (*Fagopyrum tataricum*) is an important crop used for edible food and medicinal usage. Drought annually brings reduction in crop yield and quality, causing enormous economic losses. Transcription factors are often involved in the regulation of plant responses to environmental stresses. In this study, we identified 233 MYB transcription factors in tartary buckwheat and classified them into 13 groups, including 1R, R2R3, 3R, 4R types. Gene structure and conserved motifs of these 233 *FtMYBs* suggested the relative conservation of these FtMYBs within each group. There is strong collinearity within the genomes of *F. tataricum*, with identifying syntenic gene pairs of *FtMYB*. Further, the expansion of *FtMYB* genes was attributed to whole genome duplication. The enrichment analysis of *cis*-acting elements in the *FtMYB* genes indicated that *FtMYBs* may participate in abiotic stress responses. The transcriptional changes of *FtMYB* genes in tartary buckwheat were then investigated using public data and qPCR. A number of *FtMYB* genes exhibited apparent transcript levels in the detected tissues and most of them disturbed their expression after the treatment of PEG6000 or natural treatment of tartary buckwheat seedlings. Some of the *FtMYB* genes showed a similar expression trend with qPCR validation. *FtMYB* gene *FtPinG0005108900.01* were shown to activated by PEG6000 and natural drought treatment, and its encoded protein localizes to nucleus, revealing it as a typical transcription factor. Overexpression of *FtPinG0005108900.01* increase the drought tolerance, and transcriptome analysis indicated that lignin synthesis other than flavonoid biosynthesis pathway was activated in the overexpressing plants following drought treatment. Our results provided detailed evolution and comparative genomic information of *FtMYBs* in tartary buckwheat and dissected the function of a *FtMYB* gene *FtPinG0005108900.01* in response to drought.

**Supplementary Information:**

The online version contains supplementary material available at 10.1186/s12870-024-06019-y.

## Introduction

Tartary buckwheat (*Fagopyrum tataricum*, *F. tataricum*) is an important crop widely cultivated for medicinal and edible usage. It contains a wide range of nutrients including bioactive components such as polyphenols, phytosterols, vitamins, carotenoids, and minerals, all which endow tartary buckwheat with various health benefits [1, 2]. There are a total of 21 species in the genus of *Fagopyrum* from the germplasm collection in China [3], and two of them, *F. esculentum* (*F. esculentum*, common buckwheat) and *F. tataricum*, are widely cultivated for food or medicinal usage [3]. Although these two species are closely related, large differences are identified in drought tolerance and in the contents of rutin and flavonoid [4, 5]. Attempts have been made to understand the causal genetic basis of these differences [6–8], however, much detail about specific genes involved in the drought tolerance or flavonoid content of *F. tataricum* need to be elucidated. *F. tataricum* exhibited as a drought-tolerant crop compared with *F. esculentum* [4], but there is very limited report on the molecular mechanism of the drought tolerance by *F. tataricum*.

Drought causes enormous loss in crop yield annually [9–11]. In response to low moisture in soil, plants alter the different physiological aspects to adapt to it, including root growth and architecture, stomatal responses, photosynthesis, the redox state, etc. [9, 12]. And the molecular regulatory mechanisms underlying the physiological processes are complicated and multifaceted. After the drought signal is perceived by the plant cell, a series of molecular responses occur including reactive oxygen species (ROS) generation or scavenge, Ca^2+^ ion perturbation and phytohormonal changes to cope with the coming drought threat [13]. Transcription factors (TFs) play central roles during this process spanning the drought perception, regulation of genes involved in ROS homeostasis, modulating the biosynthesis or metabolism of phytohormones, and signal transduction of phytohormones [14]. As an important hormone participating in plant drought responses, abscisic acid (ABA) regulates the stomatal movement of drought-stressed plants [15], and was suggested as a key target for drought tolerance improvement [16]. A large amount of genes is regulated by ABA [17, 18], and around 10% of these ABA-responsive genes are related to transcription, with most of them belonging to the major classes of TFs comprising of bZIP, AP2/ERF, MYB, NAC, WRKY, etc. [19–21].

MYB transcription factors are involved in different aspects of plant physiological processes, including plant growth and development, metabolism, responses to biotic and abiotic stresses [22]. A total of 196, 185, and 197 members of MYB TFs have been identified in *Arabidopsis*, *Oryza sativa*, and *Populus trichocarpa*, respectively [22]. Whereas, up to 680 members have been obtained in *Brassica napus*, a allotetraploid crop, through bioinformatic analysis [23], indicating the drastic expansion of MYB TFs in polyploid species. MYB proteins are characterized by the highly conserved MYB DNA-binding domain, which generally includes about 52 amino acids with up to four imperfect amino acid sequence repeats (R) [22]. Each MYB domain forms three α- helices, with the second and third helices of each repeat building a helix-turn-helix (HTH) structure [22]. MYB proteins can be divided into four types based on the number of repeats (one, two, three or four repeats), 4R-MYB, 3R-MYB, 1R-MYB/MYB-related, R2R3-MYB, among which R2R3 type has the most members in plants [24]. Various members of MYB proteins have been reported to participate in regulation of plant defenses against biotic or abiotic stresses [25, 26]. The R2R3 type MYB transcription factor *TaPIMP1* positively regulates the responses of wheat to *Bipolaris sorokiniana* and drought treatment [27]. BOS1, an R2R3 MYB transcription factor, is required to restrict the spread of another necrotrophic pathogen, *Alternaria brassicicola*, as well as for the tolerance to water deficit, salinity, and oxidative stress [28]. It is reasonable to dissect the particular roles *MYB* genes in different crops.

In this study, we performed genome-wide identification of *MYB* genes in tartary buckwheat, and classified FtMYBs proteins based on the phylogenetic relationship with each other. Then the gene structures, motif compositions, chromosomal locations, and *cis*-elements in promoters of *FtMYBs* were comprehensively analyzed. The collinearity between tartary buckwheat and different species (*Arabidopsis*, *Beta vulgaris*, *Glycine max*, *O. sativa*, *Vitis vinifera*) was investigated, further, the syntenic gene pairs of *FtMYBs* with corresponding *MYBs* of different species were obtained. The duplication modes of *FtMYB* genes in the chromosome of tatary buckwheat were analyzed to reveal the reason for the expansion of this gene family. In addition, the expression changes of *FtMYBs* in response to artificial drought conditions were analyzed using public data. Moreover, quantitative reverse transcription PCR was employed to validate the results of public data. One of these validated *FtMYB* genes, *FtPinG0005108900.01*, was selected for studying its function in response to drought stress. And the present research should provide clues for dissecting the roles of *FtMYB* genes involved in drought responses of tatary buckwheat in the subsequent research.

## Results

### Identification of MYB genes in tartary buckwheat

To identify MYB proteins in tartary buckwheat, hmmer search program (HMMER3.0) was conducted using Hidden Markov model (HMM) of the MYB domain against the tartary buckwheat protein database. After confirmation of the existence of MYB domain in the retrieved protein sequences, a total of 233 MYB proteins were identified. Among them, 164 proteins were recognized as R2R3-MYB type protein. Fifty-nine of them were classified into 1R-MYB/MYB-related protein, while eight and two were identified as 3R-MYB type and 4R-MYB type, respectively (Table S1). Most of the *MYB* genes locate at the two arms of the 8 chromosomes. The distribution of these *MYB* genes on different chromosomes displayed different patterns, with the chromosome Ft1 having the most number (51) of *MYB* genes. The Ft2 and Ft7 include 37 and 33 *MYB* genes, correspondingly, ranking them second and third. Whereas, chromosome Ft8 contains the least *MYB* genes, with only 14 members (Table S1, Fig. S1). The polypeptide length of FtMYB proteins range from 77 to 1482 amino acids (aa), and the isoelectric point (pI) and molecular weight (MW) of FtMYBs is within the scope of 4.25 to 11.36, and 8907 to 163520.6 Da, respectively.

**Fig. 1:**
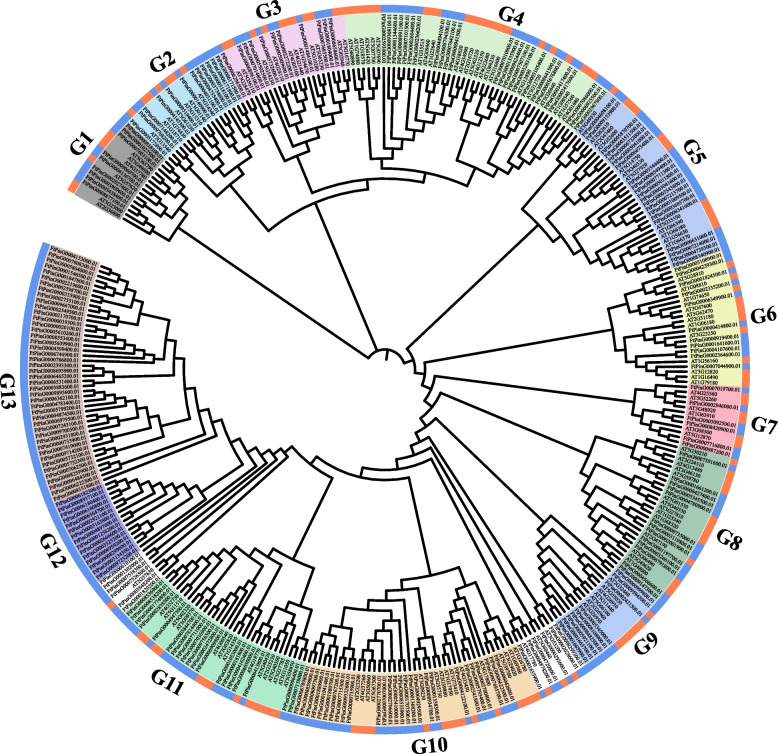
Evolutionary relationships of FtMYB. The phylogenetic relationship was analyzed using the fasttree software. The bootstrap consensus tree was inferred from 1000 replicates. The FtMYBs were divided into thirteen groups (G1 to G13) based on the clustering of the protein sequence. The proteins from *A. thaliana* and *F. tataricum* are presented in red and blue, respectively. Different groups are differentiated with different colors ad shown in the Figure

To deduce the evolutionary relationship of identified 233 MYBs, a bootstrap tree was constructed with the MYB proteins of *Arabidopsis* and tartary buckwheat. All the MYBs were classed into 13 groups (G1-G13), with each containing the number of 9, 27, 19, 15, 43, 12, 11, 24, 21, 11, 6, 13, and 9. Furthermore, 12 FtMYBs were not assigned to any group. All MYBs in G12 and G13 do not contain any MYB proteins from *Arabidopsis*, indicating an origin of these genes different from *Arabidopsis* (Fig. 1). Group 10 contains the largest number of MYBs, with 27 MYBs in this group, while group seven is the smallest group, containing only six FtMYBs.

To explore the gene structural evolution of MYBs in *F. tataricum*, all 233 *FtMYBs* were analyzed for their gene structures and conserved domains (Fig. S2 and Table S1). Through comparison of the exon numbers of *FtMYBs*, the gene structures were found to be varied, with the exon numbers from 1 to 13. There are 111 *FtMYBs* having 3 exons, occupying the largest exon types. Twenty-nine *FtMYBs* contain only one exon, and only one *FtMYB* (*FtPinG0000610100.01*) owns the greatest number of exons (13 exons) (Fig. S2 and Table S1).

The conserved domains in FtMYBs were analyzed using CD-search of National Center for Biotechnology Information (NCBI). As shown in Fig. S2, twelve principle conserved domains were identified in the 233 FtMYB proteins. MYB domain was the most prevalent domain detected in all the FtMYBs, and four different variants of MYB domain were detected, Myb DNA-binding domain, Myb_DNA-bind_6, Myb_DNA-bind_4, and Myb_Cef.

Various *cis*-acting elements and corresponding transcription factors participate in different aspects of plant physiological processes including growth and development, responses to condition stimuli, etc. The predominant 20 *cis*-acting elements detected in *FtMYBs* are shown in the Fig. S3, and Unnamed__4 is the most detected (1189 in number) *cis*-element, followed by MYC (445), MYB (444), G-box (345), and ABRE (315).

### Collinearity and gene duplication mode analysis of *MYB* genes

**Fig. 2:**
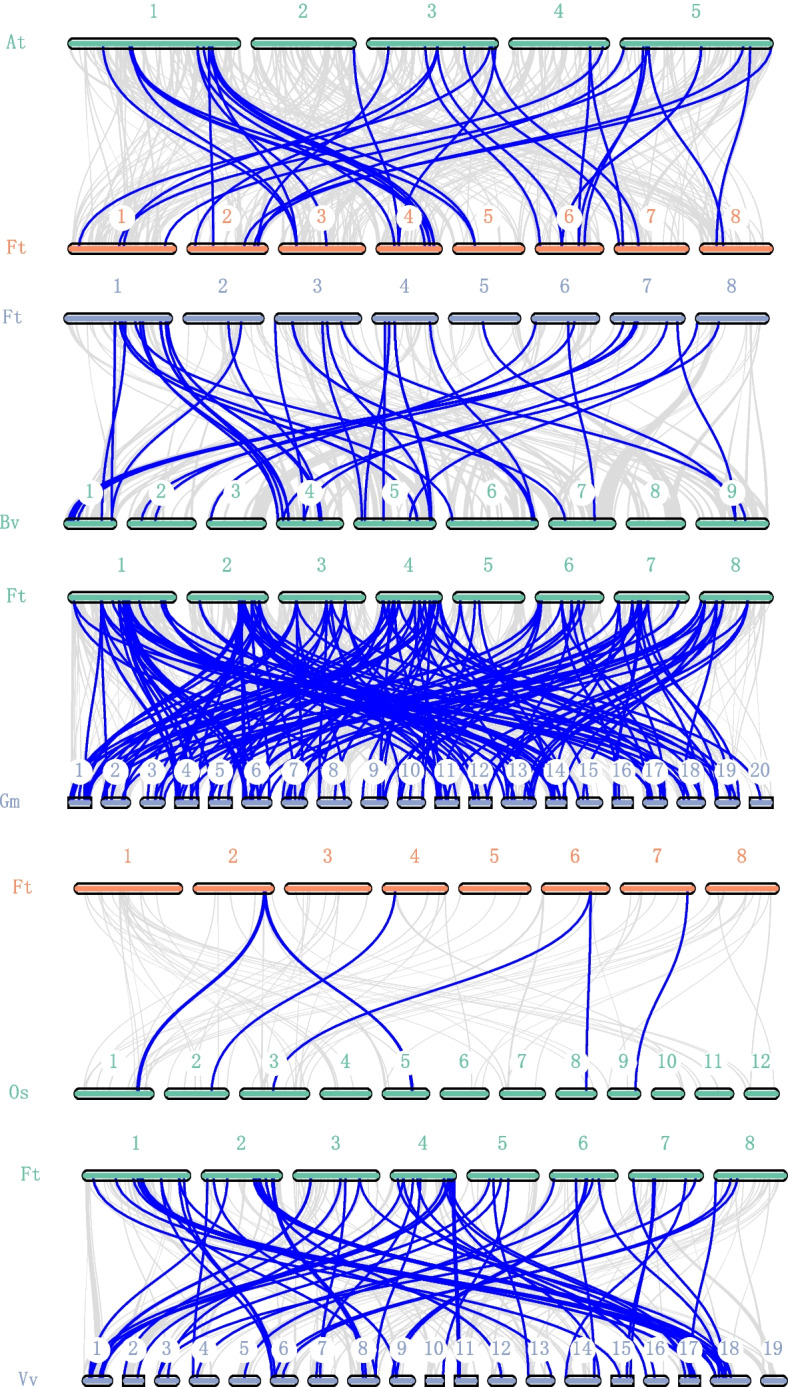
Collinearity analyses of *MYB* genes between the tartary buckwheat and the other five representative plant species. Gray lines represent the collinear blocks between the genomes of tartary buckwheat and other species, with blue lines highlighting syntenic *MYB* gene pairs. The “At”, “Ft”, “Bv”, “Gm”, “Os”, and “Vv” represent the species *A. thaliana*, *F. talaricum*, *B. vulgaris*, *G. max*, *O. sativa*, and *V. vinifera*, respectively. And the number above each chromosome indicate the chromosome number of each species

To deduce the syntenic relationship of *MYB* gene family between tartary buckwheat and other five species including *Arabidopsis*, *B. vulgaris*, *G. max*, *O. sativa*, and *Vitis vinifera*, five typical syntenic comparative diagrams were constructed, and as shown in Fig. 2, different pairs of syntenic *MYB* genes were detected in different species. In total, 46 (*A. thaliana*), 40 (*B. vulgaris*), 187 (*G. max*), eight (*O. sativa*), 64 (*V. vinifera*) syntenic *MYB* gene pairs were identified between tartary buckwheat and different species. Among the five species, the highest number of syntenic gene pairs was observed between tartary buckwheat and *G. max*, with the syntenic genes distributed throughout all chromosomes of two species (Fig. 2). In the syntenic relationship of tartary buckwheat and *Glycine max*, *FtPinG0005610200.01.T01* and *FtPinG0008553400.01.T01* were recognized to be collinear with seven different genes in soybean.

**Fig. 3:**
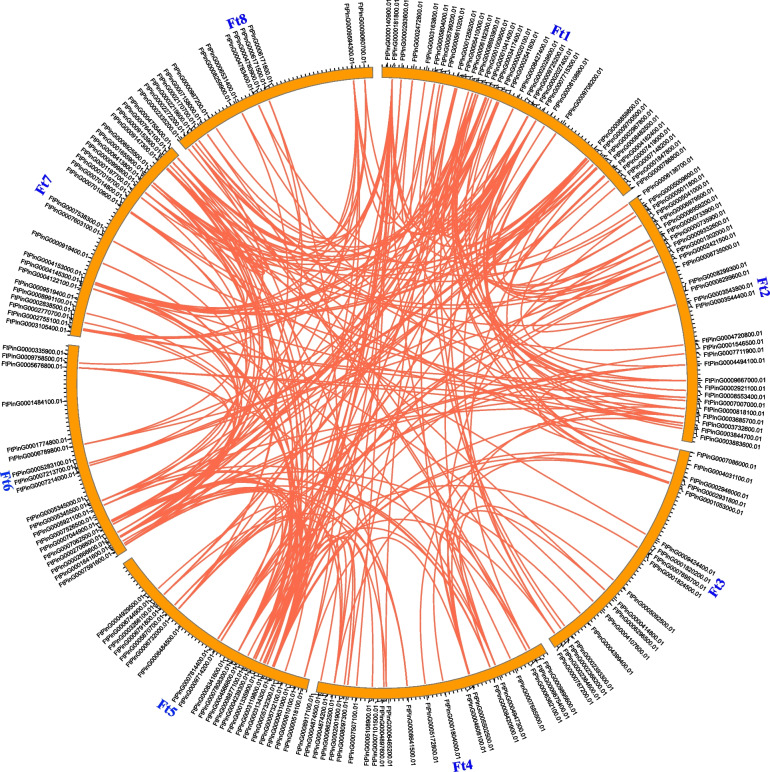
The collinear gene pairs of *FtMYB* genes on *F. talaricum* chromosomes. Red lines were used to indicate the link between a pair of syntenic genes. Each chromosome was represented using “Ft” followed by its ID, and the ID of collinear gene was marked around the position of the corresponding chromosome

Different modes of gene duplications including whole genome duplication (WGD), tandem, proximal and dispersed are considered to be the drive for gene family production [29, 30], and investigation of the duplication of a gene family may provide insight into the expansion of them. For *FtMYB* in buckwheat, four duplication models were detected in 233 *FtMYB* genes, including 156 of them produced by WGD, 47 of them assigned to dispersed duplication, 19 generated by tandem duplication, and 11 of them classified to proximal duplication (Table S1). In summary, most of the *FtMYBs* were generated by WGD, including 128 *R2R3-MYBs*, 21 *1R-MYBs*, 6 *3R-MYBs*, and 1 *4R-MYBs* (Table S1). In addition, the collinearity analysis revealed 250 collinear gene pairs within the *F. tataricum* chromosome (Fig. 3), suggesting the gene expansion fingerprint of *FtMYB* genes.

### Analysis the expression of *FtMYB*s in response to drought treatment

**Fig. 4:**
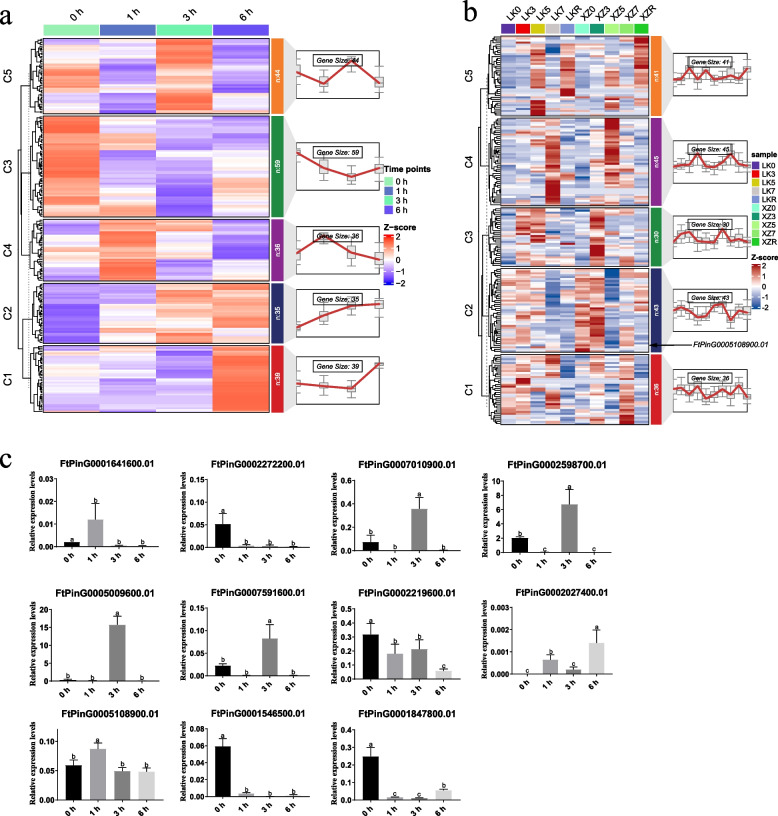
Transcriptional changes of *FtMYB* genes in tartary buckwheat after treatment with PEG6000 or natural drought stress. **a** Expression profiles of *FtMYB* genes in tartary buckwheat with treatment of PEG6000. The data was downloaded from public dataset (SRA datasets, PRJCA003569). The cultivar treated in this research is Jinqiao No. 2. The 213 *FtMYB* genes with obvious expression were classified into five clusters, C1-C5, based the expression pattern. The number containing in each cluster was indicated at the right side of each cluster. Each cluster was placed beside with a line showing the expression pattern. The samples were collected at 4 different time points (0 h, 1 h, 3 h, 6 h) after treatment. **b** Expression profiles of *FtMYB* genes in two tartary buckwheat cultivars under natural drought stress: the drought-tolerant genotype XZSN and the drought-sensitive genotype LK3. The data for this analysis was obtained from a publicly available dataset (SRA datasets, PRJNA859365). A total of 195 *FtMYB* genes were categorized into five clusters (C1-C5) using the K-means clustering method based on their expression patterns. The number of genes in each cluster is indicated on the right side of each cluster, with a line graph depicting the overall expression trend for each group. Samples XZ0, XZ3, XZ5, XZ7, LK0, LK3, LK5, and LK7 correspond to XZSN and LK3 plants subjected to drought stress for 0, 3, 5, and 7 days, respectively. XZR and LKR represent samples of XZSN and LK3 that were rewatered after drought treatment. **c** Evaluation of expression changes in *FtMYB* genes in response to PEG6000 treatments. Quantitative real-time PCR was used to determine *FtMYB* gene expression levels, with FtH3 serving as the internal control. The data are presented as mean ± SE from three biological replicates (*n* = 3)

*MYB* gene family has been reported to play crucial roles in plants’ responses to diverse abiotic stresses [31]. To investigate the expression changes of *FtMYB* genes in response to drought treatment, RNA-sequencing data related with the responses of tartary buckwheat to drought tolerance from Sequence Read Archive (SRA, PRJCA003569) was obtained for analysis. Two hundred and thirteen *FtMYB* genes displayed expression in at least one sample. As shown in Fig. 4a, the 213 *FtMYB* genes were clustered into five clusters according to the expression changes among 12 samples of 4 treatments. Cluster one (C1) included 39 genes, most of which increased the transcript levels 6 h post treatment with PEG6000 (Fig. 4a). While in cluster two (C2), most of the 35 genes increased their expression after 3 and 6 h treatment. There are 59 genes in cluster three (C3), with most of them showing decreasing expression level at different time points (Fig. 4a). Cluster four (C4), containing 36 *FtMYBs*, exhibited an expression peak after 3 h post-treatment and a return to normal afterwards (Fig. 4a). In cluster five (C5), most of the *FtMYB* genes which appeared decreased their expression 1 h post treatment with PEG6000, increased their expression after 3 h treatment, while displaying a declined trend in transcript level after 6 h treatment (Fig. 4a).

Beyond the artificial drought conditions using PEG6000, it is crucial to analyze the responses of *MYB* family genes to soil-drought environment in tartary buckwheat. We retrieved the RNA sequencing data from NCBI (PRJNA859365) and analyzed the expression level of *FtMYB* genes. A total of 195 *FtMYB* genes exhibited expression across 30 samples from two tartary buckwheat genotypes, LK3 (drought-sensitive genotype) and XZSN (drought-tolerant genotype) under 5 treatments, with 38 *FtMYB* genes showing no expression. The expression patterns of the 195 genes were categorized into five clusters. Cluster one (C1) including 41 genes, peaked in expression 5 days post-treatment, and decreased after 7 days of treatment, but increased again expression upon rewatering in both genotypes (Fig. 4b). Cluster two (C2), containing 45 *MYB* genes, peaked in expression 7 days post-treatment in drought-sensitive genotype LK3 but reached peak expression in the drought-tolerant genotype XZSN 5 days post-treatment (Fig. 4b). Cluster three (C3), consisting of 43 *MYB* genes, showed an expression peak after 5 days of drought treatment in XZSN, whereas LK3 exhibited a comparatively lower expression level at the same time point (Fig. 4b). Cluster four, also comprising 43 genes, showed higher expression level at 0 day and 3 days post-treatment in XZSN, but relatively lower levels in LZ3 under the same conditions (Fig. 4b). In cluster five, most genes increased their expression level after 3 days of treatment, decreased after 5 days’ treatment, and then recovered at 7 days post-treatment (Fig. 4b).

To validate these results, qualitative reverse transcription PCR were employed on samples with different treatments. As shown in Fig. 4c, most of the genes selected exhibited differential expression across samples with different time period treatments of PEG6000; six *FtMYBs* including *FtPinG0001641600.01*, *FtPinG0007010900.01*, *FtPinG0002598700.01*, *FtPinG0005009600.01*, *FtPinG0007591600.01*, *FtPinG0002027400.01*, and *FtPinG0005108900.01* increased their expression at different time points post treated compared to untreated samples, and four *FtMYBs* containing *FtPinG0002272200.01*, *FtPinG0002219600.01*, *FtPinG0001847800.01*, and *FtPinG0001546500.01* displayed a declining expression trend after treatment (Fig. 4c). These data suggested that *FtMYBs* responded drastically in the seedlings of tartary buckwheat treated with PEG6000.

### Overexpression of *FtPinG0005108900.01* increases the drought tolerance in *Arabidopsis*

**Fig. 5:**
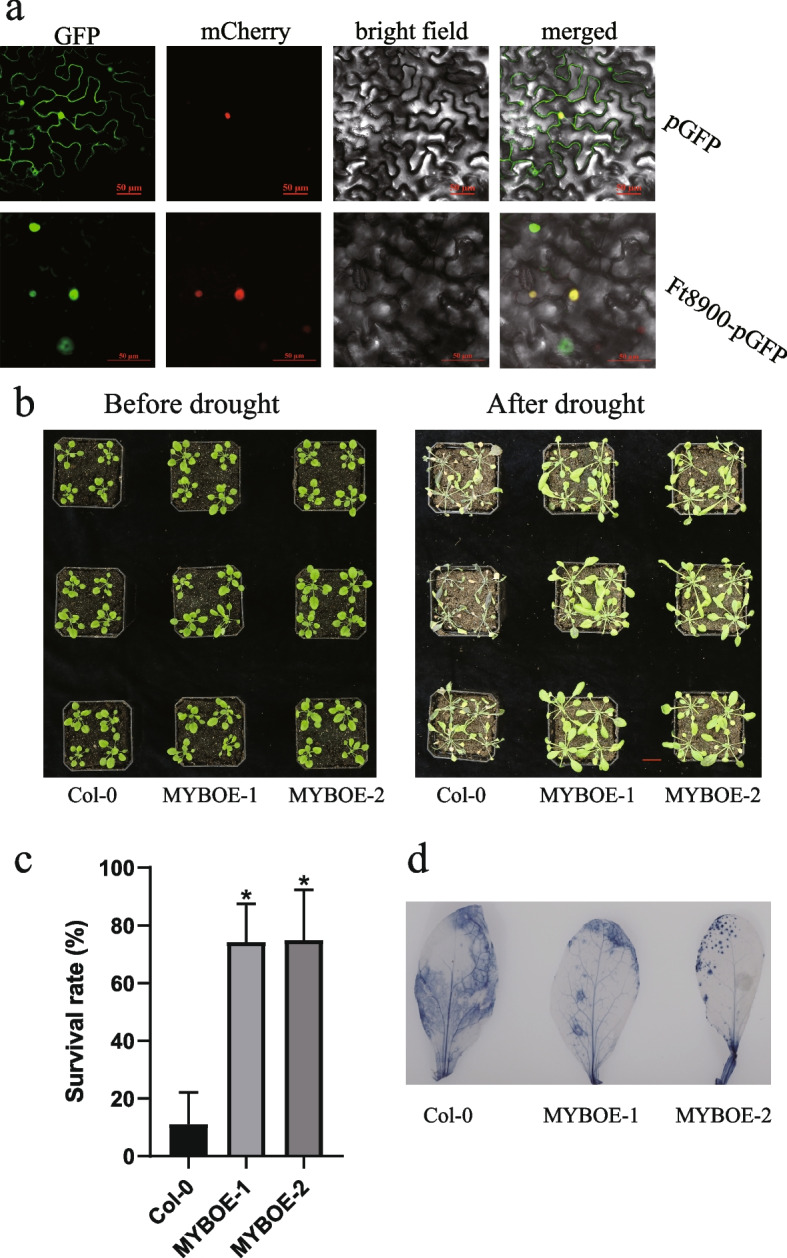
Overexpression of *FtPinG0005108900.01* increase the drought tolerance of *Arabidopsis*. **a** Subcellular localization assay of the FtPinG0005108900.01 protein. The pGFP and Ft8900-pGFP constructs were transiently expressed in tobacco leaves. The GFP indicates fluorescence from GFP channel, and the mCherry indicates the mCherry signal. Bright field represents the signal scanned under bright field. Results were observed with confocal microscopy. Scale bars = 50 μm. **b** Visual phenotypes of FtMYBOE (MYBOE-1, MYBOE-2) and wild-type (WT) *Arabidopsis* plants before and after drought treatment. Scale bar = 2 cm. Three biological repeats were performed. **c** Survival rate of FtMYBOE and WT *Arabidopsis* plants after drought treatment. **d** NBT staining of MYBOE and WT plants after drought treatment

As the *FtMYB* gene *FtPinG0005108900.01* displayed higher expression level in the drought-resistant genotype XZSN than those in the drought-sensitive genotype LK3 through the whole process of soil-drought treatment (Fig. 4b), we speculated that *FtPinG0005108900.01* might play important roles in the responses of tartary buckwheat to drought. *MYB* genes, as transcription factors, often localized in the nucleus to regulate their target genes at transcription level. In this study, the plasmid containing CDS region of *FtPinG0005108900.01* fused with green fluorescence protein (GFP), under the control of 35S promoter, was constructed, and the signal of GFP was used for tracking the protein of FtPinG0005108900.01. As shown in Fig. 5a, GFP was observed using fluorescence microscopy, localizing FtPinG0005108900.01 in the nucleus, co-localization with the mCherry signal from marker gene (*H2B*). In contrast, the control construct, which did not contain any inserted gene, displayed GFP signal throughout the whole cell (Fig. 5a). To gain insights into the putative functions of *FtMYB* genes in the drought responses of plants, we generated *Arabidopsis* lines overexpressing *FtPinG0005108900.01* using *Agrobacterium*-mediated floral dip transformation. Two lines with higher expression levels were selected for drought treatment assays. As shown in Fig. 5b, the *FtPinG0005108900.01*-overexpressing lines (FtMYBOE) did not exhibit significant differences compared to the wild type (WT, Col-0) under normal conditions. However, FtMYBOE plants demonstrated enhanced drought tolerance, evidenced by their healthier appearance and higher survival rates compared to WT plants, which showed more severe wilting and lower survival rates (Fig. 5b, c). Additionally, to further evaluate the growth status of WT and *FtPinG0005108900.0*1-OE plants under drought stress, NBT staining were used to evaluate the levels of O^2−^ in leaves. After drought treatment, the staining of WT plants’ leaves was greater than that of *FtPinG0005108900.0*1-OE plants (Fig. 5d). This finding suggested that the overexpressing lines may accumulate of O^2−^ under drought stress conditions.

### Transcriptome analysis of *FtPinG0005108900.01* enhanced drought tolerance in *Arabidopsis*

To elucidate the molecular mechanisms underlying the observed phenotypic differences, transcriptome analyses were performed on both FtMYBOE and WT plants under normal and drought conditions. The analysis revealed substantial alterations in gene expression profiles between the two groups under normal and drought conditions. A total of 12 RNA-seq libraries were constructed and sequenced, including three replicates of WT and *FtPinG0005108900.01*-OE plants under normal condition and three replicates of WT and *FtPinG0005108900.01*-OE plants under drought stress. After filtering, 132.07 Gb clean reads were retained. The total length of the clean reads ranged from 9, 186,418,652 bp (bases pair) to 12,580,267,978 bp among the libraries, with 88.14%-98.39% of the sequenced reads from each library mapped to the *A. thaliana* genome (Table S3).

**Fig. 6:**
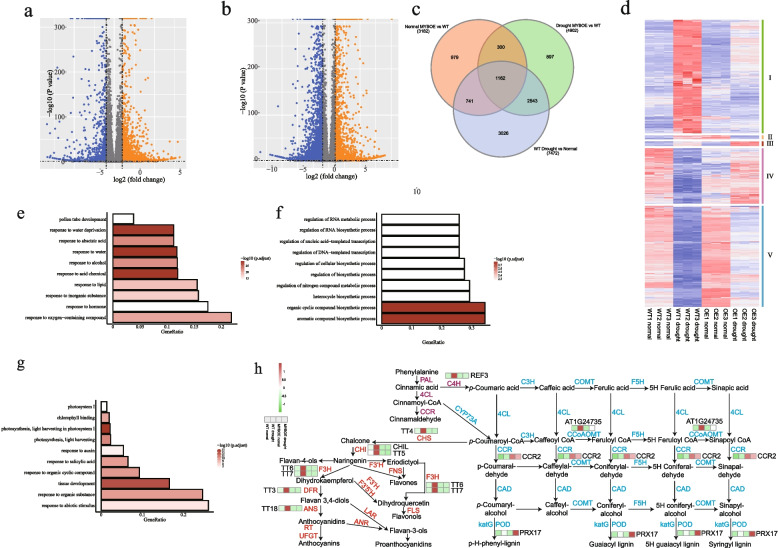
Transcriptome analysis of FtMYBOE plants in response to drought stress. Volcano plot showing significant gene expression changes (*P* < 0.05, absolute fold change > 1.0) in FtMYBOE relative to WT under normal (**a**) and drought (**b**) conditions. Orange and blue dots represent upregulated and downregulated genes, respectively (*P* < 0.05, absolute fold change > 1.0). Three biological replicates were performed for each sample at each time point. **c** Venn diagram displaying the overlap of differentially expressed genes between different groups (Normal condition MYBOE vs WT, drought condition MYBOE vs WT, drought condition WT vs WT normal condition). **d** K-means clustering of *FtPinG0005108900.01*-regulated drought-responsive genes (DRGs). **e** GO enrichment analysis of Cluster one from the k-means clustering. **f** GO enrichment analysis of Cluster three from the k-means clustering. **g** GO enrichment analysis of Cluster IV from the k-means clustering. **h** Dynamics of differentially expressed genes (DEGs) involved in flavonoid and lignin biosynthesis between FtMYBOE and WT plants under normal and drought conditions. The heat map illustrates the expression profiles of genes within the flavonoid and lignin biosynthesis pathways. Each cell in the heat map represents the expression level of a specific gene, with transcription levels indicated by TPM values, which range from green (low expression) to red (high expression). Data are presented as the mean TPM values from three biological replicates

Differentially expressed genes (DEGs) were defined as having |log2FC|> 1 (FC, fold change) and an adjusted *P*-value of < 0.05. Under normal growth conditions, significant differences in gene expression between FtMYBOE and WT plants were observed, as illustrated by the volcano plot (Fig. 6a, c, Table S3). Notably, 2232 genes were upregulated, while 950 genes were downregulated in FtMYBOE compared to WT (Table S3). Under drought conditions, the differential expression pattern became more pronounced. A total of 2092 genes were significantly upregulated in FtMYBOE plants, while 2810 genes were downregulated compared to WT plants (Fig. 6b, c, Table S3).

As the overexpression of *FtPinG0005108900.01* increased the drought tolerance of *Arabidopsis*, we focused on the changes in drought-responsive genes (DRGs) in the overexpression lines. As shown in Fig. 6c, the gene sets shared by DRGs in WT and DEGs in FtMYBOE compared to WT under drought conditions are considered DRGs affected by the overexpression of *FtPinG0005108900.01*. A total of 3705 *FtPinG0005108900.01*-induced DRGs were clustered based on their expression levels in WT and FtMYBOE lines under normal and drought conditions using K-means clustering. This analysis categorized the DEGs into five distinct clusters, each representing a unique expression pattern and potentially different functional roles in the drought responses. Cluster one comprised 1510 genes that were predominantly drought-activated but repressed in FtMYBOE lines under drought conditions (Fig. 6d). Gene Ontology (GO) enrichment analysis indicated that these genes are involved in responses to abiotic stimuli (Fig. 6e). Cluster two included 50 drought-activated genes that were upregulated under normal conditions but suppressed post-drought treatment in FtMYBOE plants (Fig. 6d). Cluster three contained 62 drought-activated genes showing enhanced expression in FtMYBOE plants compared to WT plants under drought conditions (Fig. 6d). Most of these genes were enriched in processes involved in responses to external stimuli (Fig. 6f). Cluster four comprised 737 drought-suppressed genes, whose expression was upregulated in FtMYBOE plants after drought treatment (Fig. 6d). GO analysis revealed that these genes were enriched in aromatic compound biosynthesis, jasmonic acid response, and fatty acid response processes (Fig. 6g). Cluster five included 1346 drought-suppressed genes that were inhibited in their expression in FtMYBOE plants upon drought treatment (Fig. 6d). KEGG (Kyoto Encyclopedia of Genes and Genomes) analysis of genes in Cluster one indicated that these genes were predominantly enriched in the flavonoid biosynthesis pathway, and the genes in Cluster three were found to be enriched in the phenylpropanoid biosynthesis pathway. Flavonoid biosynthesis is a branch pathway within the broader phenylpropanoid biosynthesis process, leading to the production of the largest group of polyphenolic metabolites [32, 33]. Ten *FtPinG0005108900.01*-regulated DRGs were identified to be involved in the phenylpropanoid biosynthesis (Fig. 6h). Two key enzymes in lignin biosynthesis, cinnamoyl CoA reductase encoded by *AT1G80820* and cell wall-localized class III peroxidase encoded by *AT2G22420*, were repressed in WT plants but activated in the FtMYBOE plants after drought treatment (Fig. 6h). Eight genes encoding six enzymes involved in the flavonoid biosynthesis were identified to be stimulated by the drought treatment in WT plants but did not change their expression in FtMYBOE plants (Fig. 6h).

## Discussion

More yield is required from crops grown to meet the needs of an increasing population; however, the yield of crops is often influenced by occurrences of drought and abnormal weather events all over the world. Tartary buckwheat serves as a health-benefit crop with medicinal properties, and is widely cultivated in semi-arid zones where drought frequently emerges, potentially reducing yield and quality. Recent advances reveal that transcription factors are involved in signal transduction networks from the perception of a stress signal and coordination of stress-responsive genes under abiotic stress [13, 14]. Further, different MYB transcription factors have been uncovered for their involvement in drought response in model plant *Arabidopsis* and crops [34]. It is of significance to characterize this gene family in tartary buckwheat in detail.

*MYB* genes have been identified in different plant species, with one-hundred-and-thirty *MYB* genes from *Arabidopsis* [35], 85 from rice [35], 279 in grapevine [36], 70 *R2R3-MYB* genes from sugar beet (*B. vulgaris*) [37], and 244 *R2R3-MYB* genes from *G. max* [38] recognized. MYB proteins are defined by the containing MYB DNA binding domain, and a total of 233 *FtMYB* genes, with 164 FtMYBs as R2R3-MYB type, 59 as 1R-MYB/MYB-related protein, 8 as 3R-MYb type, and 2 as 4R-MYB type, were identified in the present study. Similarly in the genome of apple and watermelon, R2R3-MYB and *1R-MYB* genes were the main *MYB* genes identified [39–41]. In addition, duplication mode of 128 R2R3-MYBs and 21 1R-MYBs of tartary buckwheat are WGD, which is also found for other gene families of tartary buckwheat [42, 43], explaining that R2R3-MYB and *1R-MYB* genes undergo expansion in the genome of tartary buckwheat. In addition, all vertebrate MYB proteins belong to the 3R-MYB subfamily [44], which also exists in most plant species, uncovering that 3R-MYB types are ancient and evolutionarily conserved shapes of *MYB* genes. Different evolution modes have been proposed for R2R3 type *MYB* genes: R2R3 type genes were evolved from an *3R-MYB* gene ancestor or from an *R1-MYB* gene by duplication [44, 45]. As the most abundant types of *MYB* genes in plants, *R2R3-MYB* genes are unique to plants [44, 46] and are thought to play a crucial role in enabling plant adaptation to terrestrial environments [47]. These genes have likely been instrumental in driving the diversification of plant species by regulating key processes such as stress response, secondary metabolism, and developmental pathways, all of which are critical for survival and reproduction in land-based ecosystems. The subgroup of R2R3-MYB genes typically exhibit similar functions [22]. In this study, R2R3-MYB are categorized into 12 subgroups with five of them not assigned to any subgroup. Three AtMYBs, AT2G23290, AT4G37260, and AT3G50060, are classified into Group 10 and exhibited similar responses to abiotic stresses [48]. Eleven FtMYBs and 12 AtMYBs are classified into Group 6, and five of the AtMYBs, AT3G23250, AT3G28910, AT1G08810, AT3G47600, AT5G62470 were identified to involve in the responses of plants to abiotic stresses [49–53], moreover, the investigated FtMYB gene *FtPinG0005108900.01* in present study belong to group 6, indicating that the same subgroup of *R2R3 MYB* genes might share similar functions.

Considering the gene structures, protein domains and phylogenetic relationships of FtMYBs, it was found that the *FtMYBs* of the same group exhibit similar exon/intron numbers or proteins domain pattern. For example, the evolutionarily close *FtMYBs*, *FtPinG0007814400.01.T01*, *FtPinG0003844700.01.T01*, *FtPinG0004947300.01.T01*, *FtPinG0002838500.01.T01*, *FtPinG0008536900.01.T01*, *FtPinG0001774800.01.T01*, *FtPinG0008538200.01.T01* have more exons/introns, and are enriched in DnaJ domains as well. All these proofs suggest that the phylogenetic analysis of FtMYB proteins in the present research is reliable. The MYB domain is responsible for binding to conserved motif of target genes, whereas the non-MYB regions often contribute to the sequence diversity and functional specificity of MYB proteins [54]. Notably, the R3 repeat within MYB domain has been identified as a key factor influencing the interaction between R/B-like BHLH proteins and 1R MYB proteins [55]. Millard et al. [54] further revealed a conserved motif critical for mediating interactions between MYB TFs from subgroup 12 and their bHLH partners. And this conserved motif plays a crucial role for the functions of corresponding MYB proteins. Therefore, the identification of conserved domain within MYBs is key for elucidating their interactions with other proteins, which is essential for regulation of complex physiological process in plants. DRE (drought-responsive element, GCCGAC) and ABRE (ABA-responsive element, ACGTG) are key cis-acting elements involved in drought responsiveness. We did not find any association between the number of these two cis-acting elements and the expression patterns of the corresponding *FtMYB* genes. This suggests that the drought response of *FtMYB* genes involves a complex regulatory network. Additionally, we observed that 111 *FtMYB* genes contained multiple DREB (dehydration-responsive element binding protein) or ABRE cis-element indicating that some *FtMYB* genes might be regulated by different DREB or ABRE binding proteins. These findings highlight the complexity and diversity of *FtMYB* gene regulation under drought conditions.

External stresses such as high salinity, drought, and extreme temperatures restrict the development and growth of crops to a certain extent or kill some crops directly, which greatly reduce the food supplies or quality [56, 57]. Tartary buckwheat is a drought tolerant crop [58], and the dissection of respective mechanism should provide clues for improving the resistance of other crops. MYB transcription factors play important roles in the responses of plants to environmental stresses [31, 34]. Here, most of the 213 *FtMYB* genes were discovered to disturb their expression after the treatment of PEG6000 in tartary buckwheat, suggesting the pivotal role of these *FtMYB* genes in the physiological responses’ regulation. In *Arabidopsis*, *AtMYB2* (*AT2G47190*) functions as transcriptional activators in ABA-inducible gene expression under drought stress [59]. Similarly, the homologous gene in tartary buckwheat *FtPinG0001197700.01* was also induced by the artificial drought condition of PEG6000 treatment. In addition, *AtMYB41* (*AT4G28110*) regulates the transcriptional and metabolic responses of *A. thaliana* to osmotic stress, and accordingly, the homologous genes in tartary buckwheat, *FtPinG0003732600.01* and *FtPinG0005009600.01*, exhibit inducement by PEG6000 treatment in the transcript level. Furthermore, 46 collinear gene pairs were detected between *Arabidopsis* and tartary buckwheat, which may suggest that the responses of *Arabidopsis* and tartary buckwheat are conserved with regard to MYB transcription factors.

In this study, we explored the role of FtPinG0005108900.01, a member of the MYB transcription factor, in enhancing drought tolerance in *A. thaliana*. Our results indicate that protein encoded by FtPinG0005108900.01 localizes to the nucleus, corroborating its function as a transcription factor. Overexpression of *FtPinG0005108900.01* in *Arabidopsis* led to significantly improved drought resistance, characterized by healthier plant phenotypes and higher survival rates compared to WT plants. To unravel the molecular mechanisms underlying this enhanced drought tolerance, we conducted a comprehensive transcriptome analysis between *FtPinG0005108900.01*-overexpressing plants and WT plants under both normal and drought conditions. Interestingly, drought-induced genes related to abiotic stress responses were not significantly upregulated in the FtMYBOE plants, suggesting a reduced perception of drought stress in these plants. This observation implies that FtMYBOE plants might experience less severe drought stress compared to WT plants. Moreover, while flavonoid biosynthesis was activated in WT plants following drought treatment, it remained unaffected in FtMYBOE plants. Anthocyanins, a key class of flavonoids [60], are known to act as co-substrates for antioxidant enzymes such as superoxide dismutase (SOD, EC1.15.1.1) and catalase (CAT, EC1.11.1.6), which scavenge excess ROS produced during drought conditions [61]. The increased anthocyanin biosynthesis in WT plants is likely a crucial self-protective response to oxidative stress [62]. In contrast, FtMYBOE plants seem to avoid this oxidative burst, possibly due to better drought adaptation. Additionally, genes involved in lignin biosynthesis were induced in FtMYBOE plants but not in WT plants. Lignin is known to enhance drought tolerance, as demonstrated in various species [63–65], and lignin-based hydrogels have been shown to mitigate drought stress in crops like maize [66]. Furthermore, genes associated with jasmonic acid, fatty acid responses were downregulated in WT plants under drought stress but upregulated in FtMYBOE plants. This differential gene expression suggests that these pathways contribute to the enhanced drought tolerance observed in FtMYBOE plants. Jasmonic acid and fatty acids are pivotal in stress responses, and their modulation may enhance resilience under drought conditions [67, 68]. Future research will focus on identifying the direct targets of FtPinG0005108900.01 and elucidating its interactions with other molecular components in the drought response network. Additionally, investigating the potential crosstalk between FtPinG0005108900.01 and other signaling pathways, particularly those involving lignin biosynthesis, jasmonic acid and fatty acids, could provide deeper insights into its role in drought tolerance. Understanding these mechanisms will be crucial for developing strategies to enhance drought resistance in crops through genetic engineering or breeding programs.

## Materials and methods

### Plant materials and growth condition

The Tartary buckwheat variety, Jiujiangkuqiao (kindly gifted by Meiliang Zhou from Institute of Crop Sciences, Chinese Academy of Agricultural Sciences), was selected for PEG6000 treatment. Seeds were surface sterilized with a 30% bleach solution, and washed four times with sterile distilled water, then germinated on wet filter papers. The uniformly-germinated seeds were moved onto rolls of papers soaked with Hoagland solution for growing until seedlings stretched out two true leaves. At this stage, the uniform seedlings were treated with 20% PEG6000. Samples were collected at 0 h, 1 h, 3 h, and 6 h, and flash-frozen in liquid nitrogen. Three biological replicates were included in each treatment, and at least 4 seedlings were sampled in each biological replicate.

### RNA extraction and quantitative reverse transcription PCR

Total RNA from different treatments was extracted using total RNA purification kit (Bioman, BIO9202). The purified total RNA was used for performing the DNA-elimination step and the first-strand cDNA synthesis using HiScript II Q RT SuperMix (Vazyme, R223) following the manufacturer’s instructions. The qPCR assay was performed using ChamQ Universal SYBR qPCR Master Mix (Vazyme) on CFX96 Real-time System (Bio-Rad, Hercules, CA, USA). The amplification programs were set as the following: initial denaturation 95 °C for 30 s, then 40 cycles of 95 °C for 10 s, 60 °C for 45 s, followed by 95 °C for 10 min. Melting curve analysis was performed by increasing 0.5 °C at 5 s/step from 67 to 95 °C. The expression levels were estimated using the 2^−ΔΔCt^ method [69] with *FtH3* gene as an internal control [70]. All the primers used for qPCR are listed in Table S2.

### Identification and phylogenetic analysis of FtMYBs

The Hidden Markov Model (HMM) file of the MYB domain (PF00249) was downloaded from the Pfam protein family database (http://xfam.org). FtMYBs were identified using HMMER 3.0 [71] against the protein sequence database of tartary buckwheat (http://mbkbase.org/Pinku1/). The existence of MYB domain in candidate genes was confirmed by CD-search [72]. The MYB proteins in *Arabidopsis* were extracted according to the same procedure. The identified MYB protein sequences of tartary buckwheat and *A. thaliana* were used for multiple sequence alignments using the ClustalW program. Phylogenetic tree was generated using alignment results with fasttree [73] using a bootstrap replication of 1000.

### Chromosomal location, duplicated type, and collinear blocks analysis

Chromosomal position information for *FtMYB* genes was extracted from genome annotation of tartary buckwheat (http://mbkbase.org/Pinku1/). The *FtMYB* genes were marked around the position of the corresponding chromosomes using TBtools software [74]. MCscanX [75] was employed to analyze the gene duplication types and collinearity relationships of *FtMYBs*. The collinearity within the *FtMYB* gene family was investigated using the program ‘detect_collinearity_within_gene_families.pl’ incorporated into MCscanX. Then collinear gene pairs within *FtMYB* gene family were plotted onto the chromosome with Circos software [76]. The program ‘duplicate_gene_classifier’ was used to detect the five duplication types including WGD/segmental duplication events in the genome of *F. tataricum*, and duplication information of the *FtMYB* gene family was extracted for analysis.

### Analysis of intron–exon structure and protein conserved domains in the *FtMYB* gene family

The exon–intron structure information of *FtMYB* genes was extracted from the *F. tataricum* genome, and displayed using TBtools software [74]. The NCBI Batch CD-search was utilized to analyze the conserved domains of the FtMYBs proteins with default parameters and screened in the Excel software, keeping one of the duplicated domains locating at the close positions of the same protein.

### Prediction of *cis*-elements in the promoter of *FtMYB* genes

The detailed information of 2000 bp length promoter for the *FtMYB* genes was separated from the genome annotation of *F. talaricum*, and the corresponding sequences were extracted with Seqtk software [77]. The obtained sequences were submitted to predict the *cis*-elements in the website of PlantCare (http://bioinformatics.psb.ugent.be/webtools/plantcare/html/).

### Transcript analysis of *FtMYB* genes in response to PEG6000 treatment based on published RNA-seq data

To investigate the expression profiles of *FtMYB* genes in response to PEG6000, SRA files were downloaded from SRA database (PRJNA859365). This dataset explored the transcriptome changes of sprouted seeds of a widely cultivated Tartary buckwheat variety, Jinqiao No. 2 under the treatment of PEG6000. Besides, another dataset (SRA, PRJNA859365), illustrating transcriptomic changes of two contrasting drought-resistant Tartary buckwheat genotypes under nature drought treatment in the reproductive stage, was introduced to characterize the expression changes of *FtMYB* genes in this study. All reads files were checked for quality control and filtered using fastp software [78]. Expression of *FtMYB* genes was quantified using the software package Kallisto [79]. The transcripts per kilobase of exon model per million mapped reads (TPM) values were extracted and used for heat maps construction with pHeatmap software package [80].

### Subcellular localization of FtPinG0005108900.01

Open reading frame of *FtPinG0005108900.01* was inserted into pGreen-NsGFP to construct a GFP-tagged subcellular localization vector (Ft8900-pGFP). The empty vector without any insert was used as negative control (pGFP), and the positive control (H2B-mCherry) was prepared by fusing *H2B* gene with mCherry. The ready plasmids were transformed into *Agrobacterium* individually, and the obtained stains were then transiently expressed in the *Nicotiana benthamiana* leaves through infiltration. The transiently expressed fluorescence signal was visualized using a confocal microscope (Leica TCS SP8).

### Phenotypic analysis of *FtPinG0005108900.01* transgenic plants under drought stress

*FtPinG0005108900.01* were inserted into pCAMBIA1302 vector, driven by CaMV 35S promoter, and were transformed into *A. thaliana* Col-0 by *Agrobacterium*-mediated transformation using the floral dip method. Expression levels of *FtPinG0005108900.01* were determined across different lines using qPCR. Two lines (MYBOE-1, MYBOE-2) with higher expression levels were selected for phenotypic test. The seeds of WT (Col-0) and overexpression lines were sown in the nutrient soil and maintained in a growth chamber with 16 h light/8 h dark and temperature 22–23 ℃ for 5 weeks. The healthy plants were imposed to natural drought until phenotypic differences were observed. The survival status of different lines was recorded.

### RNA-seq and data analyses

MYBOE-1 and WT plants under normal and drought conditions were collected for RNA preparation and subsequent transcriptome analysis. Total RNA was extracted using TRIzol reagent (Invitrogen, USA), and RNA-seq libraries were prepared using the KCTM Stranded mRNA Library Prep Kit (#DR08402, Seqhealth, China) following the manufacturer’s instructions and sequenced using the HiSeq X10 sequencing platform (Illumina). The transcriptome data was stored in the National Center for Biotechnology Information (NCBI) (BioProject ID: PRJNA1179514). The obtained reads were mapped to *A. thaliana* genome (www.arabidopsis.org) using HISAT2 [81]. All mapped reads were assessed by featureCounts (Subread-1.5.1; Bioconductor) using chromosome sequences and genome annotation file. Then transcripts per million (TPM values) were calculated. The differentially expressed genes (DEGs) between transgenic and WT plants were identified using the Deseq2 package [82], with thresholds of false discovery rate (FDR) < 0.05 and absolute log2FC (fold change) > 1. Gene Ontology (GO) and KEGG enrichment analysis for DEGs was implemented using clusterProfiler packages [83] with a threshold of *P* value < 0.05.

## Conclusion

In conclusion, this study provides comprehensive insights into the evolution and functional roles of MYB transcription factors in tartary buckwheat (*F. tataricum*), particularly in response to drought stress. We identified and categorized 233 *FtMYB* genes, highlighting their structural conservation and genomic synteny, which suggest a strong evolutionary foundation for their roles in stress responses. The analysis of *cis*-acting elements and expression profiles further supports the involvement of these FtMYBs in abiotic stress adaptation. Specifically, the *FtMYB* gene *FtPinG0005108900.01* was found to play a pivotal role in enhancing drought tolerance, with its overexpression triggering the lignin biosynthesis pathway under drought conditions. These findings not only expand our understanding of *FtMYB* gene evolution but also present potential targets for improving drought resilience in tartary buckwheat through genetic engineering.

## Supplementary Information

Supplementary Material 1: Table S1. The information of the *FtMYB* gene family in *B. napus*.

Supplementary Material 2: Table S2. Information of primers used in this study.

Supplementary Material 3: Table S3. Summary of all samples sequencing data and differential expression analysis results.

Supplementary Material 4: Fig. S1. Locations of the *FtMYB* genes in the chromosomes of *B. napus*. The chromosome number is indicated at the bottom of each chromosome.

Supplementary Material 5: Fig. S2. Phylogenetic relationships, gene structures and the conserved protein motifs of the *FtMYB* genes. a. Exon-intron structures of the tartary buckwheat *MYB* genes. The phylogenetic tree was constructed based on the coding region sequences of tartary buckwheat *MYB* genes. The black lines in the right Fig. indicate the full length of the corresponding FtMYBs genomic DNA, and the red rectangles denote the UTR, the blue rectangles represent the exons of *FtMYB* genes. Black lines between exons are introns. b. Conserved protein motifs of *MYB* genes in tartary buckwheat. The conserved motifs are identified with CD-search and displayed in different colored boxes.

Supplementary Material 6: Fig. S3. The most abundant *cis*-elements identified in the promoters of *FtMYB* genes in tartary buckwheat. The number above each column denotes the number each *cis*-element detected by the PlantCare software.

## Data Availability

Transcriptome data that support the findings of this study have been deposited in the National Center for Biotechnology Information (NCBI) with accession number PRJNA1179514.
