# Supplementary material for: Analysis of the MYB gene family in tartary buckwheat and functional investigation of FtPinG0005108900.01 in response to drought
Source: BMC Plant Biol. 2025 Jan 7;25:25. doi: 10.1186/s12870-024-06019-y (PMC11706168; doi:10.1186/s12870-024-06019-y)
Supplement: Supplementary file 4 — Supplementary Material 4: Fig. S1. Locations of the FtMYB genes in the chromosomes of B. napus. The chromosome number is indicated at the bottom of each chromosome. [file 12870_2024_6019_MOESM4_ESM.pdf]

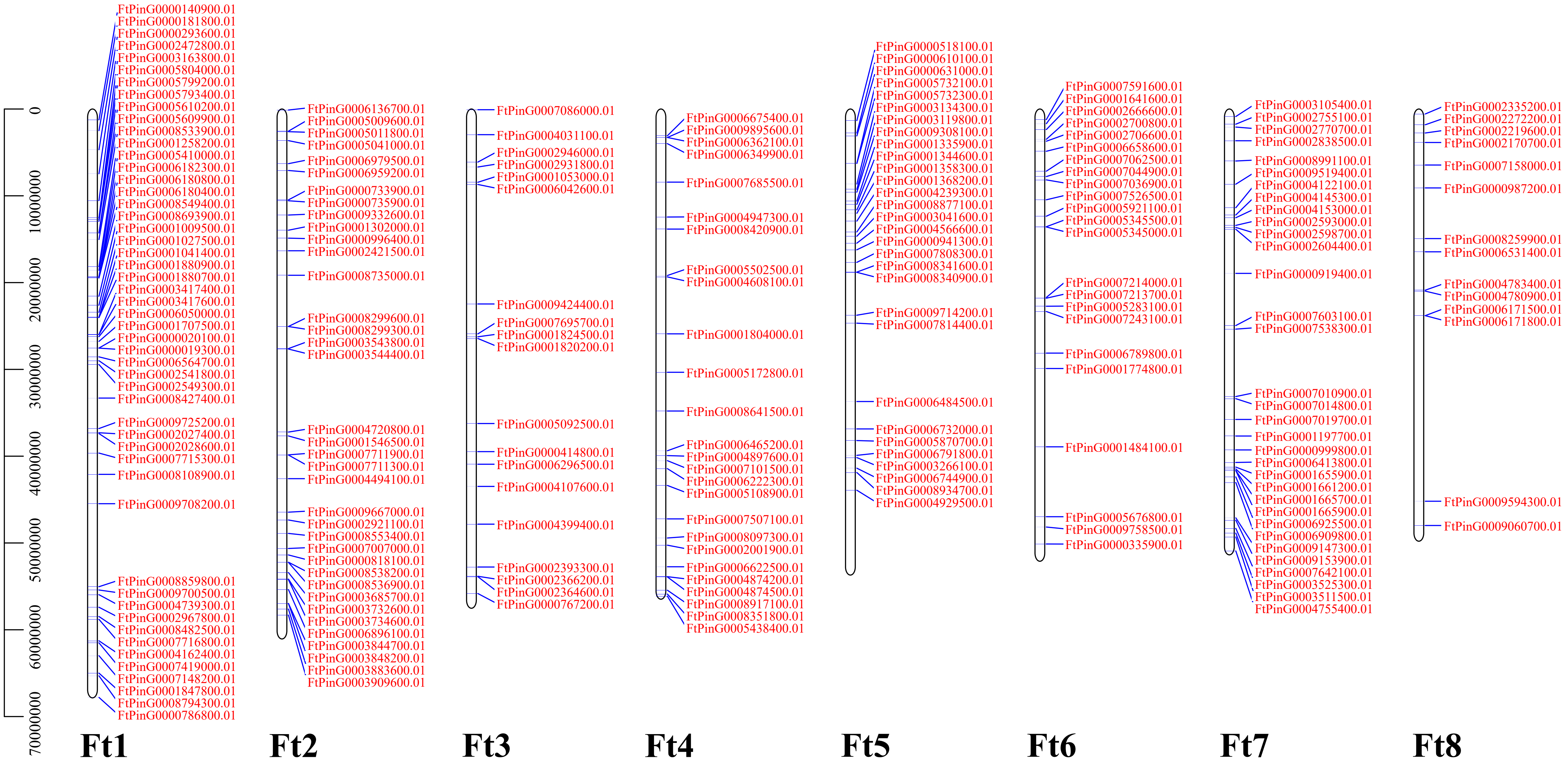

Fig. S1. Locations of the *FtMYB* genes in the chromosomes of *B. napus*. The chromosome number is indicated at the bottom of each chromosome.
