# Supplementary material for: Analysis of the MYB gene family in tartary buckwheat and functional investigation of FtPinG0005108900.01 in response to drought
Source: BMC Plant Biol. 2025 Jan 7;25:25. doi: 10.1186/s12870-024-06019-y (PMC11706168; doi:10.1186/s12870-024-06019-y)
Supplement: Supplementary file 5 — Supplementary Material 5: Fig. S2. Phylogenetic relationships, gene structures and the conserved protein motifs of the FtMYB genes. a. Exon-intron structures of the tartary buckwheat MYB genes. The phylogenetic tree was constructed based on the coding region sequences of tartary buckwheat MYB genes. The black lines in the right Fig. indicate the full length of the corresponding FtMYBs genomic DNA, and the red rectangles denote the UTR, the blue rectangles represent the exons of FtMYB genes. Black lines between exons are introns. b. Conserved protein motifs of MYB genes in tartary buckwheat. The conserved motifs are identified with CD-search and displayed in different colored boxes. [file 12870_2024_6019_MOESM5_ESM.pdf]

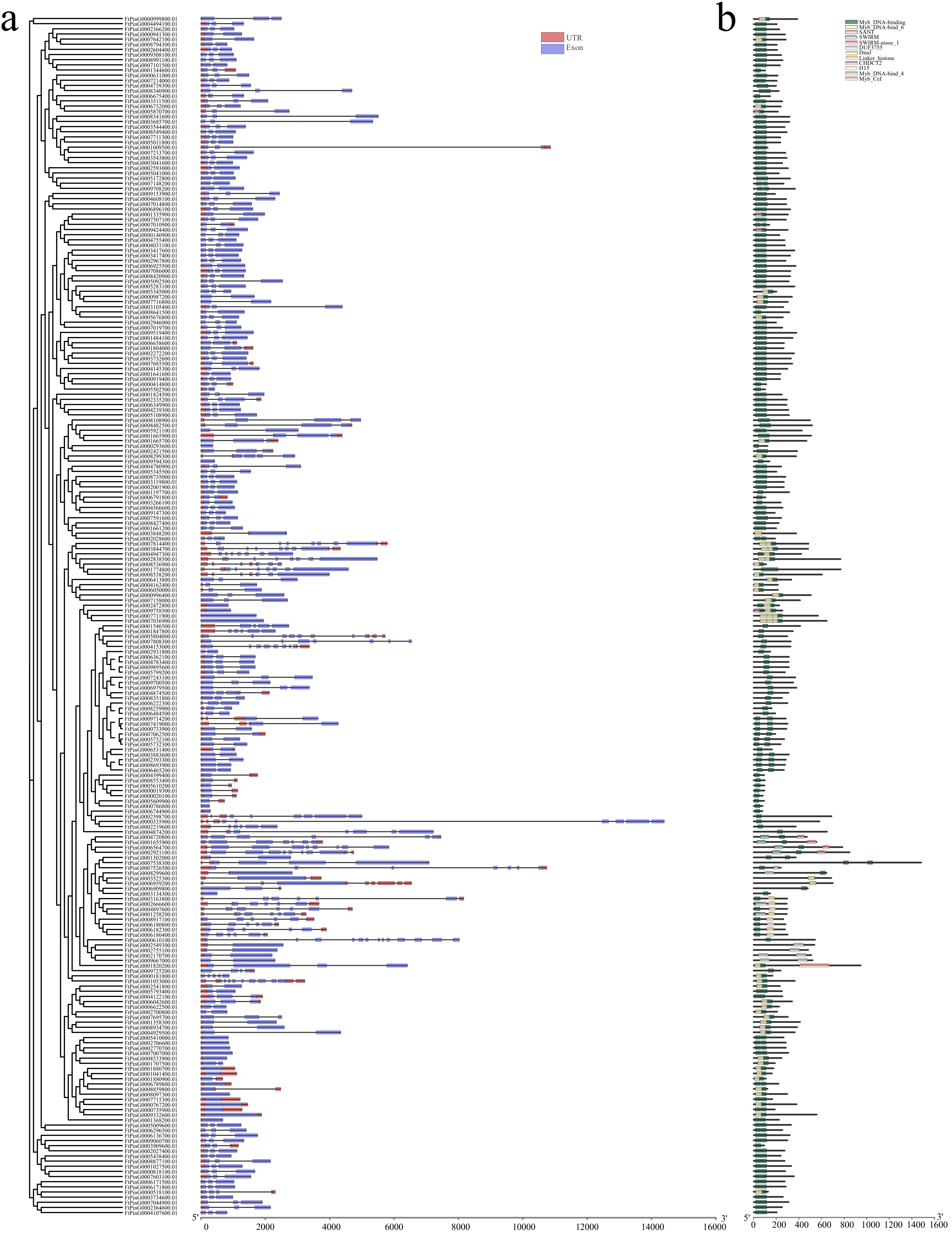

Fig. S2. Phylogenetic relationships, gene structures and the conserved protein motifs of the *FtMYB* genes. a. Exon-intron structures of the tartary buckwheat *MYB* genes. The phylogenetic tree was constructed based on the coding region sequences of tartary buckwheat *MYB* genes. The black lines in the right Fig. indicate the full length of the corresponding *FtMYBs* genomic DNA, and the red rectangles denote the UTR, the blue rectangles represent the exons of *FtMYB* genes. Black lines between exons are introns. b. Conserved protein motifs of *MYB* genes in tartary buckwheat. The conserved motifs are identified with CD-search and displayed in different colored boxes.
