# Supplementary material for: Analysis of the MYB gene family in tartary buckwheat and functional investigation of FtPinG0005108900.01 in response to drought
Source: BMC Plant Biol. 2025 Jan 7;25:25. doi: 10.1186/s12870-024-06019-y (PMC11706168; doi:10.1186/s12870-024-06019-y)
Supplement: Supplementary file 6 — Supplementary Material 6: Fig. S3. The most abundant cis-elements identified in the promoters of FtMYB genes in tartary buckwheat. The number above each column denotes the number each cis-element detected by the PlantCare software. [file 12870_2024_6019_MOESM6_ESM.pdf]

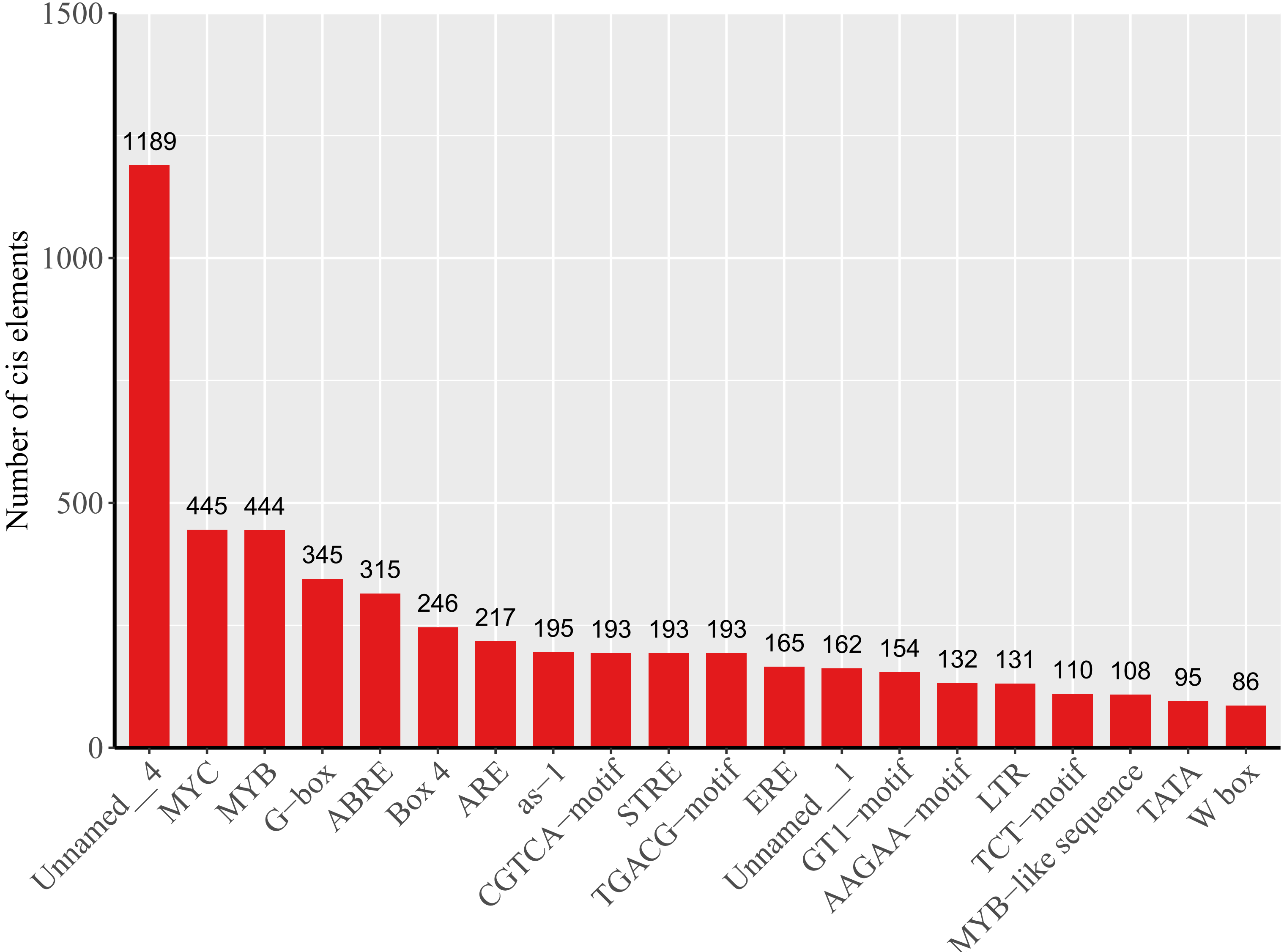

Fig. S3. The most abundant cis-elements identified in the promoters of *FtMYB* genes in tartary buckwheat. The number above each column denotes the number each cis-element detected by the PlantCare software.
